# Multi-Layered Analysis of TGF-β Signaling and Regulation via DNA Methylation and microRNAs in Astrocytic Tumors

**DOI:** 10.3390/ijms26167798

**Published:** 2025-08-12

**Authors:** Klaudia Skóra, Damian Strojny, Dawid Sobański, Rafał Staszkiewicz, Paweł Gogol, Mateusz Miller, Przemysław Rogoziński, Nikola Zmarzły, Beniamin Oskar Grabarek

**Affiliations:** 1 Department of Neurological Rehabilitation, District Hospital of St. Padre Pio in Sędziszów Małopolski, 39-120 Sędziszów Małopolski, Poland; 2 Collegium Medicum, WSB University, 41-300 Dąbrowa Górnicza, Poland; drstrojny.ds@gmail.com (D.S.); drdsobanski@gmail.com (D.S.); rafalstaszkiewicz830@gmail.com (R.S.); drpawelgogol@gmail.com (P.G.); nikola.zmarzly@gmail.com (N.Z.); bgrabarek7@gmail.com (B.O.G.); 3 Department of Neurology, New Medical Techniques Specialist Hospital of St. Family in Rudna Mała, 36-060 Rzeszow, Poland; 4 Department of Neurosurgery, St. Raphael Hospital, 30-693 Krakow, Poland; 5 Department of Neurosurgery, 5th Military Clinical Hospital with the SP ZOZ Polyclinic in Krakow, 30-901 Krakow, Poland; 6 Department of Neurosurgery, Faculty of Medicine in Zabrze, Academy of Silesia in Katowice, 40-555 Katowice, Poland; 7 Department of Anesthesiology and Intensive Care, Our Lady of Perpetual Help Hospital in Wołomin, 05-200 Wołomin, Poland; 8 Department of Trauma and Orthopedic Surgery, Our Lady of Perpetual Help Hospital in Wołomin, 05-200 Wołomin, Poland; 9 Pain Treatment Clinic, Our Lady of Perpetual Help Hospital in Wołomin, 05-200 Wołomin, Poland; 10 Department of Neurology, Independent Public Healthcare Institution of the Ministry of Internal Affairs and Administration in Rzeszow, 35-111 Rzeszow, Poland; mateusz.d.miller@gmail.com; 11 Dental Office, Resdent Stomatology Rogozinscy, 35-326 Rzeszow, Poland; resdent.stomatologia@gmail.com; 12 Faculty of Medicine and Health Sciences, Andrzej Frycz Modrzewski University in Cracow, 30-705 Krakow, Poland

**Keywords:** astrocytic tumors, TGF-β signaling, epigenetic regulation, DNA methylation, microRNA, SMAD proteins, MAPK1, glioma, gene expression profiling, prognostic biomarkers

## Abstract

Astrocytic tumors are a heterogeneous group of glial neoplasms characterized by marked differences in biological behavior and patient prognosis. Transforming growth factor-beta (TGF-β) signaling plays a pivotal role in astrocytoma pathogenesis; however, the extent and mechanisms of its epigenetic regulation remain poorly understood. This study aimed to investigate how promoter methylation and microRNA-mediated mechanisms regulate key genes within the TGF-β signaling pathway across various astrocytoma grades. Tumor tissue samples from 65 patients with WHO grade II–IV astrocytomas were analyzed using Affymetrix gene expression and microRNA microarrays. Promoter methylation of TGF-β signaling genes was assessed using methylation-specific polymerase chain reaction (MSP). Gene expression was validated by reverse transcription quantitative polymerase chain reaction (RT-qPCR), and protein levels were quantified using enzyme-linked immunosorbent assay (ELISA). MicroRNA targets were predicted using bioinformatic tools, and survival analyses were conducted using Kaplan–Meier and Cox regression models. Six genes—*SMAD1*, *SMAD3*, *SKIL*, *BMP2*, *SMAD4*, and *MAPK1*—showed significant upregulation in high-grade tumors (fold change > 5.0, *p* < 0.05), supported by RT-qPCR and protein-level data. Promoter hypomethylation and reduced expression of regulatory microRNAs (e.g., hsa-miR-145-5p targeting SMAD3) were more common in higher-grade tumors. Protein–protein interaction analysis indicated strong functional interconnectivity among the overexpressed genes. High protein levels of SMAD1, SMAD3, and SKIL were significantly associated with shorter overall survival (*p* < 0.001). This multi-level analysis reveals that astrocytic tumor progression involves epigenetic derepression and microRNA-mediated dysregulation of TGF-β signaling. Elevated expression of SMAD1, SMAD3, and SKIL emerged as strong prognostic indicators, underscoring their potential as biomarkers and therapeutic targets in astrocytic tumors.

## 1. Introduction

Astrocytic tumors, which arise from glial astrocytes in the central nervous system, represent a heterogeneous group of neoplasms with diverse degrees of malignancy, histological complexity, and clinical behavior. According to the World Health Organization (WHO), these tumors are graded from I to IV, with grades II–IV classified as infiltrating astrocytomas. Grade IV, known as glioblastoma (GBM), represents the most aggressive form, with a median survival of only 12–18 months despite the use of multimodal therapy [1,2,3]. Lower-grade astrocytomas (grades II and III) typically exhibit a more indolent clinical course but possess the potential for malignant transformation, thereby worsening patient prognosis over time [4].

The molecular characterization of astrocytic tumors has revolutionized their classification and clinical management. The 2016 WHO classification was the first to integrate key genetic alterations—such as IDH mutations and 1p/19q codeletion—into tumor nomenclature [5]. The updated 2021 WHO classification further prioritizes molecular stratification, refining diagnostic categories based primarily on genomic and epigenetic features rather than histology alone [6]. This paradigm shift has led to the redefinition of specific astrocytic tumor subtypes and emphasized the essential role of molecular diagnostics in guiding treatment decisions and predicting outcomes [7,8,9].

Among the most clinically relevant molecular alterations are IDH1/2 mutations, MGMT promoter methylation, 1p/19q codeletion, TP53 mutations, and EGFR amplification. Importantly, IDH-mutant astrocytomas are associated with significantly longer survival compared to their IDH-wildtype counterparts, even at higher histological grades [8,9,10]. These findings support the emerging view that tumor behavior is more accurately predicted by molecular profiles than by histological grade alone.

Transforming growth factor-beta (TGF-β) signaling plays a critical and paradoxical role in both normal central nervous system physiology and oncogenesis. The TGF-β family consists of three isoforms in humans—TGF-β1, TGF-β2, and TGF-β3—each encoded by a distinct gene but sharing high structural homology and overlapping biological functions [11]. These cytokines regulate numerous cellular processes, including proliferation, differentiation, immune regulation, and extracellular matrix remodeling [12]. In the healthy brain, TGF-β supports immune homeostasis and neuroprotection; however, in malignant contexts, it exhibits a dual role [13]. During early tumorigenesis, TGF-β acts as a tumor suppressor, but in advanced disease stages, it promotes tumor progression through immunosuppression, epithelial–mesenchymal transition (EMT), and neovascularization—collectively referred to as the “TGF-β paradox”.

In gliomas, overexpression and dysregulation of TGF-β isoforms have been linked to increased tumor invasiveness, therapeutic resistance, and poor prognosis. Furthermore, recent studies highlight that TGF-β activity within the glioma microenvironment influences not only tumor cells but also immune and stromal components, positioning TGF-β as a central modulator of tumor plasticity [14].

In recent years, epigenetic modifications have emerged as pivotal regulators of glioma development and progression [15,16]. These heritable but reversible changes in gene expression—independent of alterations in the DNA sequence—include DNA methylation, histone modifications, chromatin remodeling, and the activity of non-coding RNAs such as microRNAs (miRNAs) and long non-coding RNAs (lncRNAs) [17,18]. Epigenetic dysregulation disrupts numerous oncogenic and tumor-suppressive pathways, including TGF-β signaling, thereby contributing to gliomagenesis, immune escape, and therapeutic resistance [19].

In astrocytic tumors, aberrant DNA methylation patterns are known to influence tumor classification and clinical outcomes. For example, MGMT promoter methylation status is routinely assessed to predict responsiveness to alkylating chemotherapy in glioblastoma [20]. However, the broader implications of epigenetic regulation on key signaling pathways such as TGF-β remain insufficiently explored [21]. Enzymes such as histone deacetylases (HDACs), methyltransferases, and chromatin remodelers can repress or activate TGF-β target genes, thereby modulating proliferation, stemness, and invasive capacity [22]. Additionally, regulatory RNAs—particularly miRNAs—are capable of targeting multiple nodes within the TGF-β signaling, including ligands, receptors (e.g., TGFBR1/2), intracellular SMADs, and downstream effectors, adding further complexity to its role in glioma biology [23].

A growing body of research, including work by Naik et al. [19] and Ding et al. [23], underscores the significance of epigenetic control over TGF-β signaling as a major determinant of tumor progression and therapeutic resistance. These investigations demonstrate how non-coding RNAs and histone modifications modulate TGF-β transcriptional activity, thereby influencing key processes such as EMT, angiogenesis, and immune evasion [19,23]. Nevertheless, much of this evidence is derived from non-CNS tumor models, highlighting a critical knowledge gap regarding the specific epigenetic regulation of TGF-β signaling in astrocytic tumors.

To date, relatively few studies have systematically investigated the epigenetic regulation of TGF-β isoforms across astrocytoma malignancy grades. A notable exception is the study by Kurowska et al. [24], which offered valuable insights into the differential expression of TGF-β1, TGF-β2, and TGF-β3 isoforms in astrocytic tumors of varying malignancy. Based on microarray and RT-qPCR analyses of 43 tumor samples, the authors reported significant upregulation of TGF-β2 in high-grade tumors (G3/G4) compared to low-grade gliomas (G2), along with increased expression of TGF-β1 in the microarray dataset [24]. However, while this study confirmed transcriptional alterations of TGF-β isoforms and associated genes, it did not investigate the epigenetic mechanisms responsible for these changes. Specifically, methylation profiles, histone modifications, and non-coding RNA expression were not assessed [24]. Accordingly, the present study aimed to evaluate differences in the expression patterns of mRNAs, promoter methylation status, and microRNAs related to TGF-β signaling pathways in astrocytic tumors, with particular attention to variation across malignancy grades.

## 2. Results

### 2.1. mRNA Microarray and RT-qPCR Analysis

Out of the 110 genes associated with the TGF-β signaling pathway (KEGG hsa04350), one-way ANOVA identified 27 genes as significantly differentially expressed between grade II (G2) and higher-grade (G3/G4) astrocytomas (fold change [FC] > 1.50 or <–1.50; *p* < 0.05; Table A1). To further refine the analysis and emphasize the most biologically relevant alterations, a more stringent threshold was applied (FC > 5.0 or <–5.0; *p* < 0.05), yielding a subset of six genes with markedly altered expression: *SKIL*, *BMP2*, *SMAD1*, *SMAD3*, *SMAD4*, and *MAPK1*. Notably, *SMAD1*, *SMAD3*, and *SMAD4* exhibited consistent expression changes in both G3 and G4 tumors compared to G2. Microarray profiling revealed a clear upregulation of all six genes in G3/G4 astrocytomas relative to G2, indicating a potential enhancement of TGF-β-mediated transcriptional activity in higher-grade tumors (Table 1). In the following sections, we focus specifically on these six prominently dysregulated genes, as they serve as key effectors or modulators within the TGF-β signaling cascade and are likely to contribute critically to the molecular shifts associated with malignant progression.

**Figure 1:**
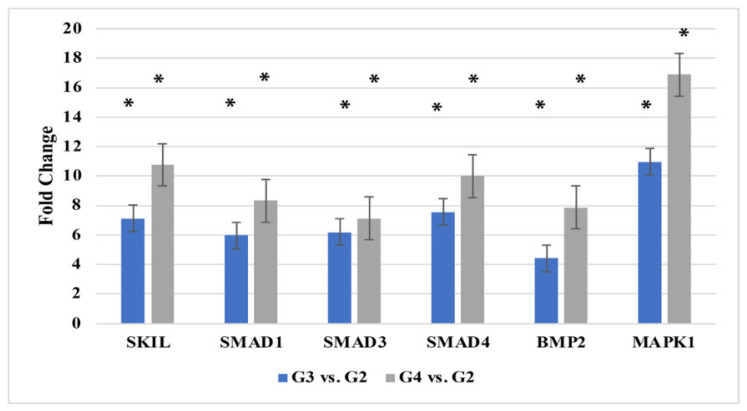
Quantitative expression analysis of six TGF-β-associated mRNAs using reverse transcription quantitative polymerase chain reaction (RT-qPCR). Data represent mean ± standard deviation (SD) from three independent experiments (*n* = 3). Statistical significance was evaluated using one-way ANOVA with post hoc testing. Asterisk indicates statistically significant differences compared to G2 (*p* < 0.05). *SKIL*, SKI-like proto-oncogene; *BMP2*, bone morphogenetic protein 2; *SMAD1*, SMAD family member 1; *SMAD3*, SMAD family member 3; *SMAD4*, SMAD family member 4; *MAPK1*, mitogen-activated protein kinase 1 (ERK2).

As shown in Figure 1, RT-qPCR analysis confirms the observed expression patterns, highlighting the consistent overexpression of selected transcripts.

### 2.2. In Silico Identification of miRNA Regulators of TGF-β Signaling-Associated Genes

To investigate the post-transcriptional regulation of TGF-β signaling components, we first performed miRNA profiling using the Affymetrix GeneChip™ (Santa Clara, CA, USA) miRNA 2.0 array on astrocytic tumor tissue samples. Subsequently, in silico prediction tools—TargetScan and miRDB—were employed to identify high-confidence miRNA–mRNA interactions corresponding to the differentially expressed TGF-β-related transcripts (Table 2). Notably, *SKIL* was predicted as a target of hsa-miR-1277-3p (target score: 100), and this miRNA was significantly downregulated in both G3 and G4 tumors (fold change: –3.18 ± 0.98 and –3.81 ± 0.12, respectively), supporting a potential derepression of *SKIL* in higher-grade tumors.

*SMAD1* and *SMAD3* were identified as putative targets of hsa-miR-30a-5p and hsa-miR-145-5p, respectively, both exhibiting strong negative fold changes in G3/G4 samples, consistent with their roles as suppressive regulators of these transcripts. Similarly, *SMAD4* was predicted to be regulated by hsa-miR-155-3p, which showed moderate downregulation in both high-grade groups.

For *BMP2*, two regulatory miRNAs—hsa-miR-587 and hsa-miR-302c-5p—were predicted, both displaying decreased expression, particularly in G4 tumors. Interestingly, *MAPK1* exhibited an inverse regulatory pattern, with significant upregulation of hsa-miR-130b-3p observed in G3 (+2.87 ± 0.37) and G4 (+3.16 ± 0.12) tumors, suggesting potential feedback or compensatory regulatory mechanisms.

### 2.3. Methylation Patterns of Selected Genes Related to TGF-β Signaling Pathway in Astrocytic Tumor Samples

**Figure 2:**
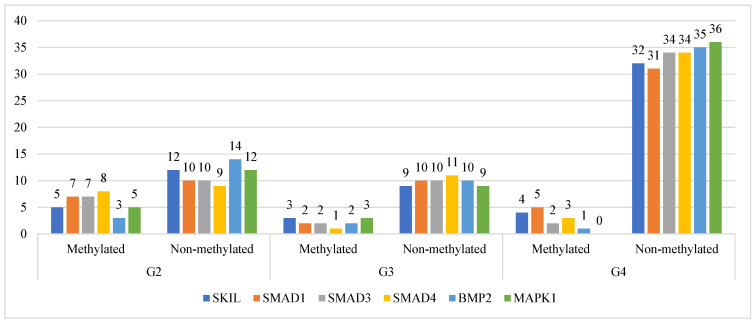
Frequency of promoter methylation in TGF-β signaling-related genes (*SKIL*, *SMAD1*, *SMAD3*, *SMAD4*, *BMP2*, and *MAPK1*) across astrocytic tumor grades (G2, G3, and G4). Methylation status was assessed using methylation-specific PCR (MSP) and interpreted in a binary manner (methylated vs. unmethylated). The histogram-like representation reflects the number of unmethylated cases per gene and grade, indicating a trend toward promoter hypomethylation with increasing tumor grade.

Figure 2 illustrates the promoter methylation status of six genes involved in the TGF-β signaling pathway—*SKIL*, *SMAD1*, *SMAD3*, *SMAD4*, *BMP2*, and *MAPK1*—across astrocytic tumors of increasing malignancy (grades G2, G3, and G4). The data reveal a clear trend toward reduced promoter methylation frequency with increasing tumor grade. Notably, *MAPK1* and *BMP2* exhibit complete or near-complete loss of promoter methylation in G4 samples, indicative of strong epigenetic derepression. *SKIL* also demonstrates a pronounced shift, with 32 out of 36 G4 cases showing a non-methylated promoter status, suggesting transcriptional upregulation driven by loss of epigenetic silencing. *SMAD3* and *SMAD4* similarly display decreased promoter methylation in higher-grade tumors; however, the degree of demethylation is more variable, implying potential heterogeneity in their epigenetic regulation. These findings support the notion that promoter hypomethylation contributes to the overexpression of key TGF-β signaling components during astrocytoma progression and may underlie their functional activation in the high-grade tumor microenvironment.

*SKIL*, SKI-like proto-oncogene; *BMP2*, bone morphogenetic protein 2; *SMAD1*, SMAD family member 1; *SMAD3*, SMAD family member 3; *SMAD4*, SMAD family member 4; *MAPK1*, mitogen-activated protein kinase 1 (ERK2).

### 2.4. Analysis of Differences in Concentration of SKIL, BMP2, SMAD1, SMAD3, SMAD4, and MAPK1 in Astrocytic Tumor Obtained via ELISA

ELISA-based quantification of selected proteins involved in the TGF-β signaling pathway revealed a significant increase in expression levels correlating with tumor grade (Table 3). Compared to G2 tumors, high-grade astrocytomas (G3 and G4) exhibited elevated levels of SKIL, SMAD1, SMAD3, BMP2, and MAPK1, with most differences reaching statistical significance (*p* < 0.05). Notably, SMAD4 showed a marked increase only in G4 tumors (Table 3).

### 2.5. Functional Enrichment Analysis (PPI) of TGF-β Signaling-Associated Genes

**Figure 3:**
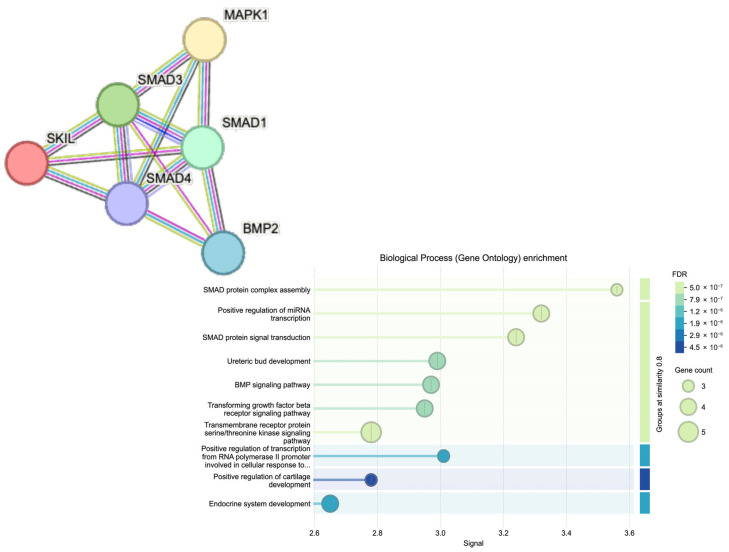
Interaction network of selected proteins generated using the STRING database.

Figure 3 presents a protein–protein interaction (PPI) network comprising six key proteins—SKIL, SMAD1, SMAD3, SMAD4, BMP2, and MAPK1—involved in the TGF-β signaling pathway. The network consists of six nodes connected by 12 edges, resulting in an average node degree of 4, indicating that each protein interacts with approximately four others. The high average local clustering coefficient (0.85) reflects a strong degree of interconnectivity, suggesting that these proteins not only engage in pairwise interactions but also form a tightly integrated functional module. As anticipated, STRING-based analysis revealed a high level of interaction confidence, consistent with well-established connections among SMAD proteins, BMP2, MAPK1, and SKIL within the TGF-β signaling cascade. Gene Ontology (GO) enrichment analysis further emphasized canonical TGF-β-related functions, including SMAD complex assembly and receptor-mediated signaling. While these results validate the biological coherence of the selected gene set, they largely reaffirm known pathway architecture rather than reveal novel interactions, as the initial selection was based on curated KEGG pathway membership.

*SKIL*, SKI-like proto-oncogene; *BMP2*, Bone morphogenetic protein 2; *SMAD1*, SMAD family member 1; *SMAD3*, SMAD family member 3; *SMAD4*, SMAD family member 4; *MAPK1*, Mitogen-activated protein kinase 1 (ERK2).

The dot plot visualizes enriched biological processes based on similarity grouping at 0.8 cutoff. Dot size corresponds to the number of genes involved, while the color intensity represents the false-discovery rate (FDR). Key enriched processes include SMAD protein complex assembly, TGF-β receptor signaling, BMP signaling, and positive regulation of transcription and miRNA expression.

### 2.6. Kaplan–Meier Survival Analysis and Cox Proportional Hazards Model for SKIL, BMP2, SMAD1, SMAD3, SMAD4, and MAPK1 in Astrocytic Tumors

**Figure 4:**
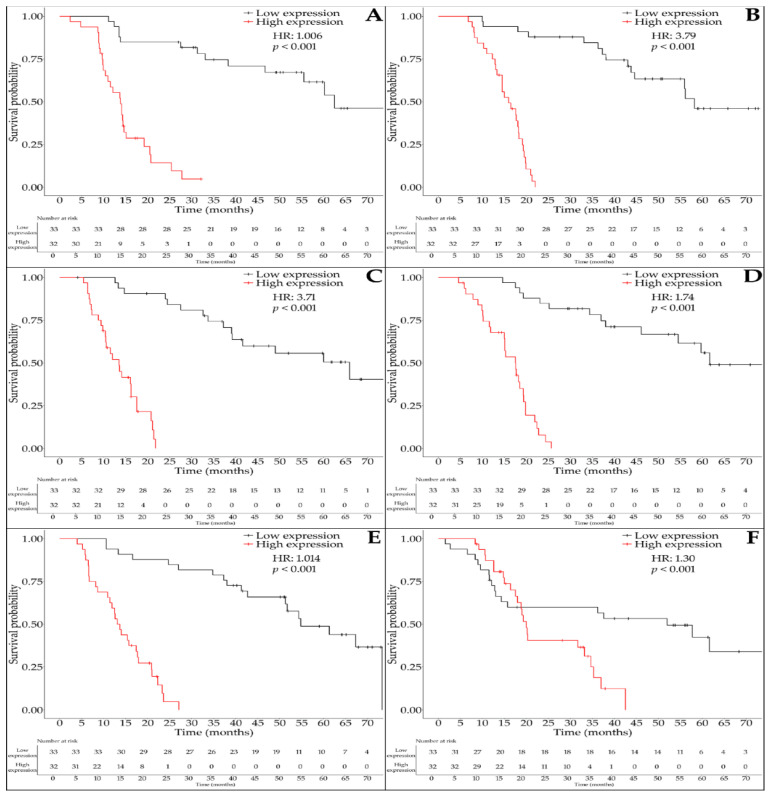
Kaplan–Meier survival curves for overall survival stratified by protein expression levels of (**A**) SKIL, (**B**) SMAD1, (**C**) SMAD3, (**D**) SMAD4, (**E**) BMP2, and (**F**) MAPK1 in patients with astrocytic tumors. *X*-axis: survival time (months). *Y*-axis: cumulative survival probability. Red lines indicate high expression; black lines indicate low expression. Number of patients at risk is displayed for all time points below each survival curve to improve transparency and interpretability.

Survival analyses revealed a consistent pattern linking elevated protein expression of TGF-β signaling components with worse prognosis in patients with astrocytic tumors. Among the examined markers, SKIL stood out as a significant negative prognostic factor. Higher SKIL levels were associated with an increased risk of death (HR = 1.006; 95% CI: 1.003–1.009; *p* < 0.001), where every 100 ng/mL increment in concentration translated to an approximately 82% increase in mortality. The Cox model fit was robust (χ^2^ = 49.49, *p* < 0.0001), and the Kaplan–Meier curve demonstrated a clear separation in survival between high and low expression groups (Figure 4A).

A similarly strong prognostic value was observed for SMAD1, with higher expression levels linked to a markedly increased risk of mortality (HR = 3.79; 95% CI: 2.21–6.48; *p* < 0.000001). The average SMAD1 concentration in the cohort was 6.06 ng/mL. The model’s excellent fit (χ^2^ = 92.22, *p* < 0.000001) and the pronounced drop in survival between 20 and 40 months underscore SMAD1’s relevance as a marker of aggressive disease (Figure 4B).

The unfavorable survival pattern extended to SMAD3, which was also significantly associated with poor outcomes. Patients with higher SMAD3 expression faced more than a threefold increase in mortality risk (HR = 3.71; 95% CI: 1.80–7.65; *p* = 0.00039), with a mean concentration of 10.63 ng/mL. The survival curve for SMAD3 mirrored that of SMAD1, with substantial divergence emerging in the 20–40 month follow-up window (χ^2^ = 88.25, *p* < 0.000001; Figure 4C).

Further supporting this trend, elevated SMAD4 levels were linked to increased mortality as well (HR = 1.74; 95% CI: 1.47–2.06; *p* < 0.000001). The average SMAD4 concentration was 5.41 ng/mL. Survival probability dropped significantly beyond 20 months in the high-expression group, as shown by the Kaplan–Meier analysis (χ^2^ = 58.50, *p* < 0.000001; Figure 4D).

This negative prognostic pattern also applied to BMP2, whose overexpression was associated with an elevated risk of death (HR = 1.014; 95% CI: 1.006–1.023; *p* = 0.0012). With a mean level of 465.54 pg/mL, BMP2’s model performance was strong (χ^2^ = 63.29, *p* < 0.000001), and survival curves showed a gradual decline in the high-expression group after the 20-month mark (Figure 4E).

Lastly, MAPK1 overexpression was significantly predictive of poor survival outcomes (HR = 1.30; 95% CI: 1.12–1.52; *p* = 0.0006). The average concentration of MAPK1 was 4.52 ng/mL, and the model remained statistically valid (χ^2^ = 13.05, *p* = 0.0003). Kaplan–Meier curves confirmed a steady decline in survival for patients with high MAPK1 levels (Figure 4F).

## 3. Discussion

The malignant progression of astrocytic tumors is driven by the cumulative dysregulation of transcriptional, epigenetic, and post-transcriptional mechanisms. In this study, we present multi-level evidence demonstrating that key mediators of the TGF-β signaling pathway—SKIL, BMP2, SMAD1, SMAD3, SMAD4, and MAPK1—are consistently upregulated in high-grade astrocytomas. This coordinated overexpression occurs at both the transcript and protein levels and is accompanied by promoter hypomethylation and suppression of regulatory miRNAs [25,26,27]. Together, these converging alterations amplify TGF-β signaling activity and are strongly associated with poor clinical outcomes, reflecting a functionally significant reprogramming of the tumor signaling architecture [28,29,30,31].

These findings expand upon our previous research, which identified epigenetic deregulation of SMAD isoforms in relation to tumor grade and prognosis. The current study broadens this framework by integrating transcriptomic, epigenetic (promoter methylation), post-transcriptional (miRNA), and protein-level data. This multi-omics approach reveals a network-wide dysregulation involving both canonical (SMAD-dependent) and non-canonical (MAPK-mediated) branches of TGF-β signaling [32].

In non-neoplastic glial cells, canonical TGF-β signaling through SMAD2/3 and SMAD4 is predominantly cytostatic and homeostatic [33,34]. However, in gliomas, this pathway is hijacked to promote tumorigenic processes such as epithelial–mesenchymal transition (EMT), immune evasion, and extracellular matrix remodeling. In our analysis, the upregulation of SMAD3 and SMAD4 was associated with increased transcriptional activation of oncogenic targets [35,36].

In addition to canonical signaling, the BMP branch—particularly BMP2, SMAD1, and SMAD4—was significantly upregulated in high-grade tumors. This finding contrasts with earlier studies that linked BMP2 signaling to cellular differentiation and favorable prognosis in glioblastoma. For example, Zhou et al. identified *BMP2* mRNA as a component of a favorable glioma grading model, with higher expression linked to lower-grade tumors and improved survival [37]. Similarly, Li et al. showed that a BMP2-mimicking peptide promoted differentiation and reduced self-renewal in glioblastoma stem-like cells [38].

This apparent contradiction may reflect the context-dependent roles of BMP signaling. While BMP2 promotes differentiation in glioma stem-like cells under specific conditions, its upregulation in bulk tumor tissue—as observed here—may instead reflect a non-functional or dysregulated compensatory response, possibly driven by an immunosuppressive or mesenchymal microenvironment. Alternatively, BMP2 expression may be a failed compensatory attempt at differentiation that is overridden by tumor-intrinsic resistance mechanisms or epigenetic silencing of downstream effectors.

These complexities highlight the need for mechanistic studies at single-cell or spatial resolution to delineate whether BMP2 exerts tumor-promoting or tumor-suppressive effects in different tumor compartments or stages. Our findings suggest that BMP2 signaling in glioblastoma does not follow a unidirectional paradigm but may exhibit dual or paradoxical roles depending on molecular subtype, tumor microenvironment, and differentiation status.

Consistent with this, SMAD1, a principal downstream effector of BMP2, was also significantly overexpressed. Although BMP signaling can inhibit growth in normal neuroepithelial cells, SMAD1 activation in gliomas has been linked to mesenchymal transformation and enhanced plasticity [39,40]. The simultaneous upregulation of SMAD1, SMAD3, and SMAD4 suggests a functional convergence between canonical TGF-β and BMP signaling arms, which may cooperatively reinforce transcriptional programs that drive stemness, invasion, and therapeutic resistance [41,42,43].

The role of SKIL in this context warrants particular attention. Traditionally considered a repressor of SMAD-mediated transcription [44,45], SKIL was consistently overexpressed in high-grade tumors and associated with poor prognosis [46]. This suggests that SKIL may act as a context-dependent oncogene, potentially modulating co-activators or chromatin accessibility in response to elevated TGF-β signaling [47]. Indeed, prior studies have linked SKIL overexpression to apoptosis resistance in other tumor types [45,48], underscoring its potential as a therapeutic target.

MAPK1, a key effector of the non-canonical TGF-β signaling axis, was also markedly upregulated in higher-grade tumors [49]. Given its role in ERK signaling and its promoter hypomethylation status, MAPK1 likely supports mitogenic and pro-survival signaling that bypasses canonical SMAD-mediated controls [50,51]. This ERK pathway activation has been previously implicated in glioma cell proliferation, radioresistance, and metabolic reprogramming [52,53,54]. Our data further support the hypothesis that the interplay between canonical and non-canonical TGF-β branches establishes a robust signaling redundancy [55,56] that sustains malignant phenotypes despite potential therapeutic blockade of individual nodes [57,58,59].

On the epigenetic level, promoter hypomethylation of BMP2, SMAD1, and MAPK1 was especially pronounced in grade IV tumors, where promoter regions were almost entirely unmethylated. This pattern reflects transcriptional derepression of genes that are typically silenced in differentiated neural tissue [60,61,62]. Such epigenetic deregulation aligns with the glioma-CpG island methylator phenotype (G-CIMP), previously associated with high-grade, biologically aggressive gliomas [63], and further supports the potential utility of DNA methylation profiling in astrocytoma stratification [64,65,66,67,68].

Post-transcriptional modulation via miRNAs further shaped the expression landscape of the TGF-β signaling [69,70]. The marked downregulation of hsa-miR-145-5p, a validated repressor of *SMAD3*, permits the accumulation of SMAD3 protein and may amplify oncogenic signaling. Similarly, hsa-miR-199a-5p and hsa-miR-125b-5p, targeting *SMAD1* and *MAPK1,* respectively, were suppressed in higher-grade tumors, facilitating overexpression of their targets. These miRNAs are known to function in neural development and glial differentiation, and their silencing may contribute to dedifferentiation and treatment resistance [71]. Additional repression of hsa-miR-29c-3p and hsa-miR-145-3p, which regulate SKIL and SMAD4, respectively, suggests a broader miRNA-mediated disruption of TGF-β signaling regulation [72,73,74]. Together, these changes represent a network-level release of inhibitory control, promoting sustained signal transduction and transcriptional output.

PPI revealed an enriched and interconnected network linking the overexpressed gene products. SMAD4 emerged as a central scaffold protein that integrates SMAD1 and SMAD3 signaling complexes [75,76]. The dual engagement of SMAD4 by both canonical TGF-β and BMP pathways highlights its functional centrality in coordinating diverse transcriptional outputs relevant to invasion, immune suppression, and therapy resistance [39]. MAPK1, positioned outside the SMAD axis, forms functional interactions with regulators of cell cycle progression and apoptosis, further reinforcing the signaling redundancy characteristic of high-grade tumors [77,78]. SKIL, while canonically a repressor, may act in gliomas through alternative protein–protein interactions not yet fully elucidated but potentially relevant to chromatin accessibility or transcriptional pausing [79]. The overall structure of the PPI network underscores the cohesive functional integration of canonical and non-canonical pathways that drive glioma pathogenesis [80].

Clinically, the prognostic impact of these molecular alterations was striking. Elevated protein levels of SMAD1 and SMAD3 were among the strongest independent predictors of poor overall survival, with hazard ratios exceeding 3.7. High SKIL expression also conferred significant prognostic disadvantage, despite its traditional classification as a transcriptional repressor [81]. These findings suggest that in the glioma context, even canonical repressors may adopt tumor-supportive roles, depending on cellular context and cofactor availability.

The clinical relevance of these proteins extends beyond prognostication. Their expression profiles could guide biomarker-driven therapeutic strategies, including combination treatments that simultaneously inhibit SMAD-mediated transcription and MAPK-driven escape pathways [82,83,84,85,86]. Although Kaplan–Meier analyses demonstrated strong associations between high expression of SKIL, SMAD1, SMAD3, SMAD4, BMP2, and MAPK1 and poor overall survival, it is important to acknowledge that these genes were also significantly upregulated in higher-grade tumors. Thus, the observed survival disadvantage may partly reflect underlying differences in tumor grade rather than purely independent prognostic effects. Future studies incorporating multivariate survival analyses, adjusted for histological grade, are warranted to dissect the relative contributions of gene expression and tumor grade to patient outcomes.

While this study provides a comprehensive, multi-level characterization of TGF-β signaling dysregulation in astrocytic tumors, several limitations must be acknowledged. First, the analysis was conducted on bulk tumor homogenates, limiting cellular resolution and precluding the discrimination of molecular alterations between tumor cells, reactive astrocytes, and non-neoplastic components such as endothelial or immune cells. Single-cell RNA sequencing or spatial transcriptomics would be required to resolve cell-type–specific expression patterns. Second, although the cohort size was sufficient for discovery-phase analyses and statistical modeling, the sample size may limit the generalizability of the findings. Therefore, validation in larger, independent, or multicenter cohorts is necessary to confirm the prognostic significance and biological robustness of the identified alterations. Third, functional validation of predicted microRNA–mRNA interactions was not performed; specifically, luciferase reporter assays and gain/loss-of-function experiments are needed to verify direct regulatory relationships, particularly for key axes such as miR-145-5p–SMAD3. Lastly, assays of downstream pathway activity—such as SMAD nuclear translocation or transcriptional reporter assays—would provide deeper mechanistic insights into TGF-β signaling activation beyond expression-level analyses. While these limitations highlight avenues for future investigation, they do not diminish the mechanistic or clinical relevance of our findings.

## 4. Materials and Methods

This study methodology was built upon the work conducted in our previous papers [32,87].

### 4.1. Patient Enrollment and Tissue Collection

Tumor tissue samples were obtained from 65 patients (35 women, 30 men) diagnosed with astrocytic brain tumors classified as grade II (G2; low-grade), grade III (G3; anaplastic), or grade IV (G4; glioblastoma), according to the WHO criteria [4]. All patients underwent elective surgical resection at one of two neurosurgical centers in Krakow, Poland: the Department of Neurosurgery at the 5th Military Clinical Hospital with SP ZOZ Polyclinic or the Neurosurgery Department of St. Raphael’s Hospital. The mean age of patients ranged from 56 to 59 years, depending on tumor grade. Female patients included 10 with grade 2, 7 with grade 3, and 18 with grade 4 tumors; male patients included 7 (G2), 5 (G3), and 18 (G4).

All surgeries were elective and performed between 8:00 and 11:00 AM. A standardized fasting protocol was implemented, with the final preoperative meal scheduled at 6:00 PM the day before surgery. Eligibility criteria included histologically confirmed astrocytoma, age ≥ 18 years, and provision of informed consent. Patients with additional primary or metastatic malignancies, urgent surgical indications, or inadequate imaging or tissue quality were excluded.

Initial tumor suspicion was based on contrast-enhanced CT and confirmed by MRI, which included T1- and T2-weighted, FLAIR, and when indicated, diffusion tensor imaging. Functional MRI and tractography supported preoperative planning for tumors near eloquent brain areas. Intraoperative tools included neuronavigation, 5-ALA-guided fluorescence, and cortical stimulation when appropriate. Final tumor grading was determined via histopathological evaluation based on WHO guidelines [7,8,9].

### 4.2. Extraction of Total Ribonucleic Acid (RNA) from Tissues

Tumor tissues were homogenized using a T18 Digital Ultra-Turrax system (IKA Poland Ltd., Warsaw, Poland). Total RNA was isolated with TRIzol reagent (Invitrogen Life Technologies, Carlsbad, CA, USA), followed by purification using the RNeasy Mini Kit (QIAGEN, Hilden, Germany). DNase I treatment (Fermentas International Inc., Burlington, ON, Canada) was used to eliminate residual genomic DNA.

RNA integrity was assessed by electrophoresis on 1% agarose gels stained with ethidium bromide. RNA concentrations were determined by measuring absorbance at 260 n.

### 4.3. Gene Expression Microarrays

A curated set of genes involved in the TGF-β signaling cascade was compiled using pathway annotations obtained from the Kyoto Encyclopedia of Genes and Genomes (KEGG), specifically through the PathCards integrative platform (hsa04350; accessed 19 January 2025), corresponding to the “TGF-beta signaling pathway.” [88]. Transcriptomic profiling was conducted using Affymetrix HG-U133A_2.0 arrays (Affymetrix, Santa Clara, CA, USA) and the GeneChip™ 3′ IVT PLUS Reagent Kit (Catalog No. 902416). Protocols were conducted according to the manufacturer’s instructions and previously described methodologies [32,87,89].

### 4.4. miRNA Profiling and Target Prediction

MicroRNA expression profiling was performed using the GeneChip™ miRNA 2.0 Array (Affymetrix, Santa Clara, CA, USA). Target prediction was performed with TargetScan (http://www.targetscan.org/) [90] and miRDB (http://mirdb.org) [91]. Predicted interactions with confidence scores >80 were considered high-confidence, whereas those with lower scores were marked for future validation [91,92].

### 4.5. qRT-PCR Validation

Gene expression changes identified by microarrays were validated using qRT-PCR with the SensiFast SYBR No-ROX One-Step Kit (Bioline, London, UK). The thermal cycling protocol consisted of an initial reverse transcription step at 45 °C, followed by polymerase activation at 95 °C for 2 min. This was succeeded by 40 amplification cycles comprising denaturation at 95 °C for 5 s, annealing at 60 °C for 10 s, and extension at 72 °C for 5 s. Gene expression levels were normalized to endogenous controls using the 2^−ΔΔCt^ method. Primer sequences are provided in Table 4.

### 4.6. DNA Methylation Assessment

Promoter CpG islands were identified using the MethPrimer tool (http://www.urogene.org/cgi-bin/methprimer/methprimer.cgi; accessed on 19 January 2025) [93]. Bisulfite conversion preceded methylation-specific PCR (MSP), performed using the QuantiTect SYBR Green PCR Kit (Qiagen GmbH, Hilden, Germany). Cycling parameters included denaturation (94 °C), annealing (65 °C), and extension (72 °C).

PCR products were resolved on 1% agarose gels stained with ethidium bromide. Fragment identification used pBR322/HaeIII markers. Methylated and unmethylated DNA controls were included (EpiTect Control DNA, Qiagen GmbH, Hilden, Germany). The characteristics of the primers designed for MSP are presented in Abbreviation (Table 5).

### 4.7. Enzyme-Linked Immunosorbent Assay (ELISA) Quantification

Quantitative assessment of selected signaling and inflammatory proteins in astrocytic tumor samples (grades G2, G3, and G4) was conducted using ELISA, strictly adhering to the manufacturers’ protocols. Each assay was performed in triplicate when sample volume allowed. Absorbance was measured at the appropriate wavelength using a calibrated microplate reader, and protein concentrations were calculated based on standard curves generated with known concentrations of the respective analytes. Protein levels were determined using commercial ELISA kits from MyBioSource Inc. (San Diego, CA, USA). The following targets were analyzed: SMAD1 (Cat. No. MBS2025476), SMAD3 (MBS161553), SMAD4 (MBS450115), SKIL (SKI-like oncogene, MBS8806352), BMP2 (bone morphogenetic protein 2, MBS137998), and MAPK1/ERK2 (mitogen-activated protein kinase 1, MBS2127197). To ensure inter-sample comparability, all protein concentrations were normalized to the total protein content of each lysate, expressed as ng/mL or pg/mL of total protein extract.

### 4.8. Statistical Methods

Data were processed using StatPlus (v. 1.1) and the Affymetrix Transcriptome Analysis Console. Normality of distributions was assessed using the Shapiro–Wilk test. Differences between groups were assessed using one-way ANOVA followed by Benjamini–Hochberg correction and Scheffé’s post hoc test. The assumption of homogeneity of variance was evaluated using Levene’s test prior to performing ANOVA. Although Scheffé’s test is relatively robust to violations of this assumption, appropriate diagnostics were conducted to confirm the validity of the statistical approach. Student’s *t*-test was used for selected pairwise comparisons. Survival analyses were performed using Kaplan–Meier curves with the log-rank test, and multivariate associations were assessed using Cox proportional hazards regression. For Kaplan–Meier survival analysis, patients were stratified into “high” and “low” expression groups using the median protein concentration as the cutoff of each biomarker. This median-based approach is widely used for exploratory biomarker analysis in oncology when no established clinical threshold exists. Survival plots were generated using R (V4.4.0) [94] and the survival (V3.8.3) [95,96] and survminer (V0.5.0) [97] packages. Based on national statistics indicating approximately 3270 new brain tumor cases in Poland during 2020–2021 [98], the cohort of 65 participants was considered acceptable. Assuming maximal variance and a 95% confidence level, the estimated margin of error was approximately 12%.

Data were processed using StatPlus (v. 1.1) and the Affymetrix Transcriptome Analysis Console. Normality of distributions was assessed using the Shapiro–Wilk test. Differences between groups were assessed using one-way ANOVA followed by Benjamini–Hochberg correction and Scheffé’s post hoc test. The assumption of homogeneity of variance was evaluated using Levene’s test prior to performing ANOVA. Although Scheffé’s test is relatively robust to violations of this assumption, appropriate diagnostics were conducted to confirm the validity of the statistical approach. Student’s *t*-test was used for selected pairwise comparisons. Survival analyses were performed using Kaplan–Meier curves with the log-rank test, and multivariate associations were assessed using Cox proportional hazards regression. For Kaplan–Meier survival analysis, patients were stratified into “high” and “low” expression groups using the median protein concentration as the cutoff of each biomarker. This median-based approach is widely used for exploratory biomarker analysis in oncology when no established clinical threshold exists. Survival plots were generated using R (V4.4.0) [94] and the survival (V3.8.3) [95,96] and survminer (V0.5.0) [97] packages. Based on national statistics indicating approximately 3270 new brain tumor cases in Poland during 2020–2021 [98], the cohort of 65 participants was considered acceptable. Assuming maximal variance and a 95% confidence level, the estimated margin of error was approximately 12%.

## 5. Conclusions

This integrative study demonstrates that astrocytic tumor progression is marked by coordinated dysregulation at the transcriptional, epigenetic, and post-transcriptional levels within the TGF-β signaling pathway. Specifically, the upregulation of SMAD1, SMAD3, SMAD4, BMP2, MAPK1, and SKIL—driven by promoter hypomethylation and reduced expression of tumor-suppressive microRNAs—leads to enhanced activation of both canonical and non-canonical branches of TGF-β signaling. These molecular alterations not only reflect the aggressive biological behavior of high-grade astrocytomas but are also significantly associated with unfavorable patient survival, highlighting their relevance as prognostic biomarkers. The findings offer a compelling rationale for the development of targeted therapeutic approaches aimed at TGF-β pathway components, potentially in combination with epigenetic or microRNA-based interventions. Future research should include functional validation and prospective, multicenter studies to translate these molecular insights into clinically applicable strategies in neuro-oncology.

## Figures and Tables

**Table 1:** Differentially expressed genes between high-grade (G3/G4) and low-grade (G2) astrocytomas identified by microarray profiling (FC > 5.0 or <–5.0; *p* < 0.05).

ID	mRNA	log_2_FC G3 Vs. G2	log_2_FC G4 Vs. G2
206675_s_at	*SKIL*	7.43 ± 0.54	9.18 ± 0.43
215889_at		7.42 ± 0.43	9.54 ± 0.76
217591_at		7.18 ± 0.87	9.71 ± 0.43
225227_at		7.21 ± 0.32	9.81 ± 0.51
210993_s_at	*SMAD1*	5.76 ± 0.87	8.18 ± 0.98
227798_at		5.87 ± 0.71	7.34 ± 1.02
205396_at	*SMAD3*	6.13 ± 0.77	7.81 ± 1.03
205397_x_at		6.21 ± 0.54	8.12 ± 0.76
205398_s_at		6.43 ± 0.91	8.43 ± 0.76
202526_at	*SMAD4*	8.16 ± 0.78	9.12 ± 0.65
202527_s_at		8.23 ± 0.60	9.87 ± 0.17
235725_at		8.43 ± 0.86	9.12 ± 1.09
1565702_at		8.11 ± 0.81	9.54 ± 1.02
1565703_at		8.71 ± 0.44	9.88 ± 1.04
205289_at	*BMP2*	5.11 ± 0.31	7.65 ± 0.87
205290_s_at		6.09 ± 0.61	7.53 ± 0.56
208351_s_at	*MAPK1*	10.11 ± 0.98	14.13 ± 1.23
212271_at		9.98 ± 1.07	14.55 ± 1.24
224620_at		9.73 ± 0.43	16.12 ± 1.32
224621_at		10.16 ± 0.76	15.65 ± 0.98
229847_at		10.54 ± 0.11	14.76 ± 1.19
1552263_at		10.17 ± 0.76	14.61 ± 1.25
1552264_a_at		10.78 ± 0.19	14.19 ± 1.28

Data are presented as mean ± standard deviation. *SKIL*, SKI-like proto-oncogene; *BMP2*, bone morphogenetic protein 2; *SMAD1*, SMAD family member 1; *SMAD3*, SMAD family member 3; *SMAD4*, SMAD family member 4; *MAPK1*, mitogen-activated protein kinase 1 (ERK2).

**Table 2:** miRNAs predicted to target TGF-β signaling components and their expression profiles in G3/G4 vs. G2 tumors.

*miRNA*	Target Score	G3 Vs. G2 (log_2_FC)	G4 Vs. G2 (log_2_FC)	Predicted mRNA Target
hsa-miR-1277-3p	100	−3.18 ± 0.98	−3.81 ± 0.12	*SKIL*
hsa-miR-30a-5p	96	−4.91 ± 0.71	−4.78 ± 0.19	*SMAD1*
hsa-miR-miR-145-5p	98	−4.18 ± 0.73	−3.91 ± 0.98	SMAD3
hsa-miR- miR-155-3p	83	−2.11 ± 0.41	−2.13 ± 0.12	*SMAD4*
hsa-miR-587	94	−3.65 ± 0.17	−4.71 ± 0.19	*BMP2*
has-miR-302c-5p	93	−2.18 ± 0.51	−3.18 ± 0.61	*BMP2*
hsa-miR-130b-3p	96	+2.87 ± 0.37	+3.16 ± 0.12	*MAPK1*

Data are presented as mean ± standard deviation. *SKIL*, SKI-like proto-oncogene; *BMP2*, bone morphogenetic protein 2; *SMAD1*, SMAD family member 1; *SMAD3*, SMAD family member 3; *SMAD4*, SMAD family member 4; *MAPK1*, mitogen-activated protein kinase 1 (ERK2).

**Table 3:** Concentration of SKIL, SMAD1, SMAD3, SMAD4, BMP2, and MAPK1 proteins in astrocytic tumor samples of different malignancy grades (G2, G3, G4), measured by ELISA.

Protein	G2	G3	G4
SKIL [ng/mL]	456.98 ± 43.91	1234.13 ± 76.12 *	1345.19 ± 51.36 *
SMAD1 [ng/mL]	2.17 ± 0.18	5.16 ± 0.61 *	8.17 ± 0.17 *
SMAD3 [ng/mL]	5.81 ± 0.34	9.18 ± 0.12 *	13.34 ± 0.13 *
SMAD4 [ng/mL]	2.15 ± 0.19	2.87 ± 0.29	7.87 ± 0.91 *
BMP2 [pg/mL]	121.12 ± 7.12	541.90 ± 19.33 *	601.19 ± 12.81 *
MAPK1 [ng/mL]	1.19 ± 0.81	5.91 ± 0.76 *	5.21 ± 2.09 *

Protein concentrations were normalized to total protein content in each sample to enable inter-sample comparison. Data are presented as mean ± standard deviation. *SKIL*, SKI-like proto-oncogene; *BMP2*, bone morphogenetic protein 2; *SMAD1*, SMAD family member 1; *SMAD3*, SMAD family member 3; *SMAD4*, SMAD family member 4; *MAPK1*, mitogen-activated protein kinase 1 (ERK2); * *p* < 0.05 vs. G2.

**Table 4:** RT-qPCR primers.

mRNA	RT-qPCR Amplification Primers (5′-3′)
*SKIL*	Forward: AGAGACTCTGTTTGCCCCAA
	Reverse: CAGGATGGGGCATTGAATGG
*SMAD1*	Forward: CACTCAACGCCACTTTTCCA
	Reverse: TCTTCAGGAGGCAGGTAAGC
*SMAD3*	Forward: CTACCAGAGAGTAGAGACAC Reverse: TCTCTGGAATATTGCTCTGG
*SMAD4*	Forward: AAAGGTCTTTGATTTGCGTC Reverse: CTATTCCACCTACTGATCCTG
*BMP2*	Forward: AATGCAAGCAGGTGGGAAAG Reverse: GCTGTGTTCATCTTGGTGCA
*MAPK1*	Forward: TGGAATAGGTTGTTTTTAAATGTTG Reverse: AAACTTTTCCTTAAACAAATCATCC
*ACTB*	Forward: TCACCCACACTGTGCCCATCTACGA Reverse: CAGCGGAACCGCTCATTGCCAATGG
*GAPDH*	Forward: GGTGAAGGTCGGAGTCAACGGA
	Reverse: GAGGGATCTCGCTCCTGGAAGA

*SKIL*, SKI-like proto-oncogene; *BMP2*, bone morphogenetic protein 2; *SMAD1*, SMAD family member 1; *SMAD3*, SMAD family member 3; *SMAD4*, SMAD family member 4; *MAPK1*, mitogen-activated protein kinase 1 (ERK2); ACTB, beta actin; GAPDH, Glyceraldehyde-3-phosphate dehydrogenase.

**Table 5:** Characteristics of primers designed for the MSP.

mRNA	M/U	NCBI Reference Sequence Database (RefSeq)	Primers (5′-3′)
*SKIL*	M	NM_005414	Forward: ATAAGGAGAATTAAAATTAAGTCGT Reverse: AATAAATAAATACAAATACCTATCGTA
	U		Forward: ATAAGGAGAATTAAAATTAAGTTGT Reverse: TAATAAATAAATACAAATACCTATCATA
*SMAD1*	M	NM_001003688.1	Forward: AGGTTTTGAGTTGTTTAGGGTAATC Reverse: ATAACATAAAACAATCCCTTCCGA
	U		Forward: AGGTTTTGAGTTGTTTAGGGTAATT Reverse: C ATAACATAAAACAATCCCTTCCAAT
*SMAD3*	M	NM_005902	Forward: TTTTTAAATTTATTTTCGAATTCGA Reverse: TAAAAAACAACCCTAAACAAAAACG
	U		Forward: TTAGATGGGTTTTTTAAGTATTTGT Reverse: ACATCCACCTCTAAATTTACTCATA
*SMAD4*	M	NM_005359	Forward: TTGGGTTAGGTGTTTTAGTGATTAC Reverse: A ACCGCCTACTACTACATCTATCGAT
	U		Forward: TTTGGGTTAGGTGTTTTAGTGATTAT Reverse: ACCACCTACTACTACATCTATCAAT
*BMP2*	M	NM_001200.4	Forward: ATTTACGAGGAAGGTTGTAATAGTC Reverse: TAAATTTAACTTAATCCAAATCGAT
	U		Forward: TTATGAGGAAGGTTGTAATAGTTGT Reverse: AAATTTAACTTAATCCAAATCAAT
*MAPK1*	M	NM_002745.5	Forward: TATCGTCGAAGTATTATTTAAGTTCGA Reverse: AAAAAAACACCGATATCTAAACACG
	U		Forward: TTGTTGAAGTATTATTTAAGTTTGA Reverse: AAAAACACCAATATCTAAACACATC

M, primers designed for methylated sequences; U, primers designed for non-methylated sequence; *SKIL*, SKI-like proto-oncogene; *BMP2*, bone morphogenetic protein 2; *SMAD1*, SMAD family member 1; *SMAD3*, SMAD family member 3; *SMAD4*, SMAD family member 4; *MAPK1*, mitogen-activated protein kinase 1 (ERK2).

## Data Availability

The data presented in this study are available in this article.

## Abbreviations

BMP	Bone Morphogenetic Protein
BMP2	Bone Morphogenetic Protein 2
CNS	Central Nervous System
CNS WHO	CNS WHO Classification
Co-SMAD	Common Mediator SMAD
CpG	Cytosine-phosphate-Guanine
DOAJ	Directory of Open Access Journals
ELISA	Enzyme-Linked Immunosorbent Assay
EMT	Epithelial-to-Mesenchymal Transition
ERK	Extracellular Signal-Regulated Kinase
FC	Fold Change
G-CIMP	Glioma CpG Island Methylator Phenotype
GO	Gene Ontology
HR	Hazard Ratio
IDH	Isocitrate Dehydrogenase
LD	Linear Dichroism
MGMT	O6-Methylguanine-DNA Methyltransferase
miRNA	MicroRNA
MSP	Methylation-Specific Polymerase Chain Reaction
mRNA	Messenger RNA
PCR	Polymerase Chain Reaction
PPI	Protein–Protein Interaction
qPCR	Quantitative Polymerase Chain Reaction
RefSeq	NCBI Reference Sequence Database
R-SMAD	Receptor-Regulated SMAD
RT-qPCR	Reverse Transcription Quantitative PCR
SKIL	SKI-like Proto-Oncogene
SMAD	Mothers Against Decapentaplegic Homolog
STRING	Search Tool for the Retrieval of Interacting Genes
TGF-β	Transforming Growth Factor-Beta
TLA	Three-Letter Acronym
WHO	World Health Organization
MAPK	Mitogen-Activated Protein Kinase
MAPK1	Mitogen-Activated Protein Kinase 1

## Appendix A

**Table A1:** Differentially expressed genes between high-grade (G3/G4) and low-grade (G2) astrocytomas identified by microarray profiling (FC > 2.0 or <–2.0; *p* < 0.05).

ID	mRNA	log_2_FC G3 Vs. G2	log_2_FC G4 Vs. G2
206675_s_at	*SKIL*	7.43 ± 0.54	9.18 ± 0.43
215889_at		7.42 ± 0.43	9.54 ± 0.76
217591_at		7.18 ± 0.87	9.71 ± 0.43
225227_at		7.21 ± 0.32	9.81 ± 0.51
210993_s_at	*SMAD1*	5.76 ± 0.87	8.18 ± 0.98
227798_at		5.87 ± 0.71	7.34 ± 1.02
205396_at	*SMAD3*	6.13 ± 0.77	7.81 ± 1.03
205397_x_at		6.21 ± 0.54	8.12 ± 0.76
205398_s_at		6.43 ± 0.91	8.43 ± 0.76
202526_at	*SMAD4*	8.16 ± 0.78	9.12 ± 0.65
202527_s_at		8.23 ± 0.60	9.87 ± 0.17
235725_at		8.43 ± 0.86	9.12 ± 1.09
1565702_at		8.11 ± 0.81	9.54 ± 1.02
1565703_at		8.71 ± 0.44	9.88 ± 1.04
205289_at	*BMP2*	5.11 ± 0.31	7.65 ± 0.87
205290_s_at		6.09 ± 0.61	7.53 ± 0.56
208351_s_at	*MAPK1*	10.11 ± 0.98	14.13 ± 1.23
212271_at		9.98 ± 1.07	14.55 ± 1.24
224620_at		9.73 ± 0.43	16.12±1.32
224621_at		10.16 ± 0.76	15.65 ± 0.98
229847_at		10.54 ± 0.11	14.76 ± 1.19
1552263_at		10.17 ± 0.76	14.61 ± 1.25
1552264_a_at		10.78 ± 0.19	14.19 ± 1.28
207530_s_at	*CDKN2B*	−2.51 ± 0.32	−4.67 ± 0.65
236313_at		−2.32 ± 0.34	−4.87 ± 0.43
202248_at	*E2F4*	2.01 ± 0.12	2.98 ± 0.19
38707_r_at		2.12 ± 0.19	3.09 ± 0.23
221586_s_at	*E2F5*	2.19 ± 0.28	3.81 ± 0.51
203304_at	*BAMBI*	3.17 ± 0.32	4.87 ± 0.43
218468_s_at	*GREM1*	2.18 ± 0.43	4.12 ± 0.32
218469_at		2.22 ± 0.65	4.18 ± 0.91
206516_at	*AMH*	2.02 ± 0.19	2.16 ± 0.13
204926_at	*INHBA*	2.87 ± 0.32	4.32 ± 0.16
210511_s_at		2.91 ± 0.31	4.51 ± 0.19
227140_at		2.71 ± 0.54	4.81 ± 0.32
205258_at	*INHBB*	3.18 ± 0.43	3.91 ± 0.39
207687_at	*INHBC*	2.11 ± 0.71	2.12 ± 0.21
203075_at	*SMAD2*	3.43 ± 0.19	4.32 ± 0.32
203076_s_at		3.32 ± 0.21	4.54 ± 0.22
203077_s_at		3.51 ± 0.32	4.32 ± 0.12
226563_at		3.76 ± 0.31	4.38 ± 0.76
235598_at		3,81 ± 0.19	4.44 ± 0.18
239271_at		3.55 ± 0.37	4.79 ± 0.23
205187_at	*SMAD5*	3.43 ± 0.43	4.31 ± 0.43
205188_s_at		3.43 ± 0.21	4.51 ± 0.32
225219_at		3.15 ± 0.81	4.48 ± 0.19
225223_at		3.81 ± 0.19	4.65±0.23
235451_at		3.45 ± 0.18	4.71 ± 0.43
207069_s_at	*SMAD6*	−4.13 ± 0.17	−4.81 ± 0.18
209886_s_at		−4.32 ± 0.31	−4.65 ± 0.43
209887_at		−4.19 ± 0.54	−4.71 ± 0.44
213565_s_at		−4.32 ± 0.43	−4.91 ± 0.23
204790_at	*SMAD7*	−3.21 ± 0.51	−4.13 ± 0.17
206320_s_at	*SMAD9*	2.12 ± 0.18	2.87 ± 0.32
227719_at		2.10 ± 0.65	2.51 ± 0.12
213044_at	*ROCK1*	3.31 ± 0.25	4.18 ± 0.19
214578_s_at		3.32 ± 0.32	4.56 ± 0.25
230239_at		3.71 ± 0.54	4.32 ± 0.19
235854_x_at		3.45 ± 0.51	4.51 ± 0.23
1561486_at		3.81 ± 0.67	4.44 ± 0.44
205596_s_at	*SMURF2*	3.12 ± 0.71	3.98 ± 0.51
227489_at		3.18 ± 0.19	4.05 ± 0.62
230820_at		3.65 ± 0.28	4.09 ± 0.71
232020_at		3.23 ± 0.54	3.98 ± 0.32
211518_s_at	*BMP4*	−3.91 ± 0.19	−4.54 ± 0.18
205430_at	*BMP5*	−3.49 ± 0.31	−3.71 ± 0.32
205431_s_at		−4.01 ± 0.29	−3.87 ± 0.47
206176_at	*BMP6*	−2.19 ± 0.71	−3.09 ± 0.61
215042_at		−2.11 ± 0.18	−3.32 ± 0.54
241141_at		−2.87 ± 0.15	−3.21 ± 0.43
209590_at	*BMP7*	−4.54 ± 0.71	−4.98 ± 0.17
209591_s_at		−4.32 ± 0.29	−4.71 ± 0.65
211259_s_at		−4.87 ± 0.34	−4.76 ± 0.23
211260_at		−4.54 ± 0.71	−4.87 ± 0.35
209747_at	*TGFB3*	2.76 ± 0.53	3.45 ± 0.46

***SKIL***, SKI-like proto-oncogene; ***BMP2***, bone morphogenetic protein 2; ***SMAD1*,** SMAD family member 1; ***SMAD3***, SMAD family member 3; ***SMAD4*,** SMAD family member 4; ***MAPK1***, mitogen-activated protein kinase 1 (ERK2); ***CDKN2B***, cyclin-dependent kinase inhibitor 2B (p15, inhibits CDK4); ***E2F4***, E2F transcription factor 4; ***E2F5***, E2F transcription factor 5; ***BAMBI*,** BMP and activin membrane-bound inhibitor; ***GREM1*,** gremlin 1, DAN family BMP antagonist; ***AMH***, anti-Müllerian hormone; ***INHBA***, inhibin subunit beta A; ***INHBB***, inhibin subunit beta B; ***INHBC*,** inhibin subunit beta C; ***SMAD2***, SMAD family member 2; ***SMAD5***, SMAD family member 5; ***SMAD6***, SMAD family member 6; ***SMAD7*,** SMAD family member 7; ***SMAD9***, SMAD family member 9 (also known as SMAD8); ***ROCK1***, Rho-associated coiled-coil containing protein kinase 1; ***SMURF2*,** SMAD specific E3 ubiquitin protein ligase 2; ***BMP4***, bone morphogenetic protein 4; ***BMP5***, bone morphogenetic protein 5; ***BMP6***, bone morphogenetic protein 6; ***BMP7***, bone morphogenetic protein 7; ***TGFB3***, transforming growth factor beta 3.
